# Development and Research of Gradient Polymer-Metal Materials Obtained Using Additive Technologies, with an Assessment of Their Functional and Mechanical Properties

**DOI:** 10.3390/polym17223014

**Published:** 2025-11-13

**Authors:** Svetlana Tyurina, Varvara Karzakova, Victor Demin, Chao Zhang, Peter Rusinov

**Affiliations:** 1 Federal State Budgetary Educational Institution of Higher Education “MIREA—Russian Technological University”, Moscow 119454, Russia; tyurina_s@mirea.ru (S.T.); v.karzakova@yandex.ru (V.K.); 2 Federal State Budgetary Institution of Science A.N. Frumkin Institute of Physical Chemistry and Electrochemistry of the Russian Academy of Sciences, Moscow 119071, Russia; barsoid@inbox.ru; 3 College of Mechanical Engineering, Yangzhou University, Yangzhou 225127, China; zhangc@yzu.edu.cn; 4 Jiangsu Key Laboratory of Surface Strengthening and Functional Manufacturing, Yangzhou University, Yangzhou 225127, China

**Keywords:** vat polymerization, chemical metallization, copper plating, additive manufacturing technologies, composite materials, functional layers

## Abstract

This paper addresses the challenge of producing lightweight, low-cost, and highly functional devices and components for the electronics industry. To tackle this issue, functionally graded materials consisting of a polymer base and a metallic conductive layer were developed. Technology for producing functionally graded materials was created and optimized. To evaluate the influence of key process parameters on the functional and mechanical properties of the composites, three-dimensional models were constructed and mathematical equations were formulated. The continuity and thickness of the surface layer were examined, the dielectric properties of the polymer material were measured, the resistance of the conductive surface layer was assessed, and adhesion tests of the surface layer were performed.

## 1. Introduction

The challenge of producing lightweight, low-cost, and highly functional devices and components is critical to modern industry. Addressing this challenge requires combining multiple materials with different properties and adapting them to various device elements or components within a simple manufacturing process. One common material combination used to achieve this goal involves metals and polymers, which are among the most frequently employed functionally graded materials [1,2,3,4,5]. These materials find applications in such fields as aerospace, automotive, instrumentation, electronics, and medicine [6,7]. Polymer materials are widely used due to their light weight, flexibility, ease of fabrication, and low cost. With the recent advances in materials science, it is essential to have materials with high strength and ductility. These mechanical properties can be achieved by using materials with a gradient structure. In the review [8], some methods for creating gradient structures, such as SMAT, SMGT, RASP, USP and Torsion, are briefly described. Using these methods, the evolution of the microstructure, strength and ductility of various materials is considered. In the work [9], the authors summarize data on gradient polymer materials with a wide range of mechanical, optical, and other properties. The term “type-one gradient materials” refers to materials with a smoothly changing elastic modulus in the range of 3–2000 MPa with no layers and interfaces, gluing, heat sealing, etc. in the material. The term “type-two gradient materials” refers to materials with a sharply changing elastic modulus and a transition zone between the glassy and highly elastic states. In the work [10], gradient materials were obtained by mixing mixtures of polyacrylic acid/sodium salt and its copolymers with acrylamide and Fe and Ce particles in an applied electric field. The authors of the article [11] developed printing strategies for multiple copper-based composites and multi-component gradient materials. This allowed us to create multiple metallic and non-metallic compounds, as well as multi-gradient materials with different compositions and structures with simultaneous gradient properties.

Additive manufacturing technologies offer significant potential for creating complex objects with multiple functions. In particular, these technologies can be used to produce embedded electronics, 3D structural electronics, conformal electronics, stretchable electronics, and more [7,12,13]. By using additive manufacturing to fabricate complex 3D geometries from multiple materials, it becomes possible to create devices that are unattainable through traditional 2D printing methods originally developed for the graphics industry. Examples include multilayer printed circuit boards, electrical connectors, 3D antennas, mission-specific satellite components, 3D structures with embedded electronics, and batteries. The field of 3D printed electronics enables low-volume production of highly complex and customizable electronic structures while reducing material waste, energy consumption, prototyping time, and costs compared to conventional methods [14,15,16,17,18,19].

The functionality of products can be enhanced by applying functional coatings to their surfaces. Currently, a wide variety of methods are used for this purpose. They include physical vapor deposition (PVD), chemical vapor deposition (CVD), spin coating, dip coating, chemical synthesis, vacuum deposition, flame and arc spraying, electrolytic deposition, chemical coating, and others. However, PVD, CVD, and thermal spray processes operate at high deposition temperatures, which can sometimes exceed the melting point of polymer substrates. Operating temperatures for CVD are approximately 200–600 °C for PVD, 800–1100 °C, while thermal spray methods can reach up to 1000 °C [20,21,22]. These methods also involve significant setup and operating costs. Additionally, the electrolytic process is unsuitable for applying coatings to non-metallic substrates [23]. In comparison, chemical vapor deposition (CVD) is a feasible option, as it can deposit layers of metals or metallic compounds onto plastics and polymers. This process offers several advantages, like uniform coating application on complex geometric contours, low deposition temperatures well below the melting point of plastics and polymers, good wear and corrosion resistance, low cost, and ability to impart electrical conductivity to the surface [24,25,26,27].

Polymer modification, including surface metallization with metallic layers, has been widely used in industry and science since its inception [28,29,30,31]. In the early years of metallization technology, plastic metallization was primarily employed to enhance decorative properties. Later, with the advancement of metallization methods, attempts were made to improve the technical properties of polymers. Consequently, plastic metallization has become increasingly prevalent in electronics and micromechanics. The development of printed circuit boards using various types of polymer materials has marked a significant breakthrough in the technological industry [32,33,34,35].

Electroless plating is a promising method for metallizing plastic 3D-printed details. However, current techniques depend on surface activation using costly metal catalysts such as palladium [36,37]. Therefore, it is essential to explore alternative solutions for metallizing polymeric materials. Electroless plating is an autocatalytic process that reduces metal ions from a solution with a chemical reducing agent. This method is highly efficient on catalytically active surfaces and does not require complex electrochemical equipment. Nevertheless, this technology relies on noble metals (Pd, Pt) as catalysts, uses highly toxic reagents during surface activation, and is unable to selectively deposit coatings without additional masking technologies [38,39,40]. Despite these drawbacks, electroless plating remains a viable option because it does not require control of current density or uniform electrode distribution. It is most commonly used to form conductive sublayers for subsequent galvanic metal deposition [41,42,43].

The metallization of polymer details produced via 3D printing presents several technological challenges due to the unique additive manufacturing characteristics. Key issues include increased surface layer porosity, pronounced anisotropy in mechanical properties, low adhesion strength at the metal-polymer interface, and unpredictable material behavior during chemical processing. As studies have demonstrated [44,45,46], the combined effect of these factors significantly degrades the quality of metallic coatings. Incorrect selection of process parameters can lead to defects such as selective metal deposition in surface imperfections, coating delamination or intense gas evolution at the interface [47,48]. These issues significantly undermine the benefits of additive manufacturing technologies, despite their exceptional flexibility and extensive shaping capabilities. Nevertheless, the combination of additive manufacturing and metallization techniques opens new prospects for rapid prototyping of functional devices.

After reviewing a number of studies [49,50,51], we decided to focus on SLA printing when developing metallization technology. It enables the production of miniature, complex-shaped components for the electronics industry.

Thus, improving metallization methods for additively manufactured details is a critical scientific and technical challenge. Addressing this challenge will broaden the application of additive technologies from experimental prototyping to mass production of functional devices.

In this study, 3D printing and chemical metallization technologies were developed. They encompass sensitization, activation, and copper plating processes. The technology utilizes volumetric resin sensitization with tannin, thereby eliminating the need for costly surface activators such as platinum and palladium, as well as avoiding the use of chemically aggressive (toxic) electrolytes.

The goal of this work is to develop composite materials with a gradient distribution of properties, combining a polymer-based material (fabricated via SLA printing) with layers of conductive metal (applied through chemical metallization). These composites demonstrate enhanced strength (compared to the polymer alone), surface-layer conductivity, and improved durability, thereby achieving the desired functional and mechanical characteristics.

## 2. Materials and Methods

The process included the following steps:

1—tannin hydration;

2—resin homogenization with a modifying additive in an emulsifying unit (AE30);

3—the resulting stable resin composition retains its properties even after storage;

4—product printing (can be a single-step process if a continuous layer is required, or a two-step process involving printing a substrate from a standard material, followed by replacing the material with a modified one to print individual structures that will be metallized);

5—exposure to an AgNO_3_ solution to create copper deposition sites on the electrolyte;

6—rinsing with distilled water, followed by drying in a specialized oven;

7—exposure to a copper plating electrolyte.

Table 1 presents the key process parameters for 3D printing and subsequent metallization used in developing the technology for producing polymer-copper materials. The composition of the electrolyte used in developing the technology for producing metallic copper layers on the polymer surface is presented in Table 2.

The layered composite material consisted of a polymer material produced via 3D printing and copper layers applied through chemical metallization. The polymer was fabricated by 3D printing of a photopolymer resin (Anycubic Water Wash, Jinhua, Zhejiang, China) combined with tannic acid (CAS 1401-55-4, China). The additive was incorporated following the procedure described below, and the resulting modified resin composition was used to 3D print the samples.

The preparation of the modified photopolymer resin involved the following steps:

- Precise weighing of tannin (accuracy ± 10^−4^ g) using a VLTE-510T (Gosmetr, St. Persburg, Russia) analytical balance;
- Gradual addition of distilled water to achieve the optimal level of hydration;
- Controlled swelling of the additive. It is essential to prevent gelatinization because it reduces homogenization;
- Thoroughly homogenize the mixture using an AE30 emulsifying unit (Shanghai Yiken Machinery Equipment Co., Ltd., Shanghai, China).

**Figure 1:**
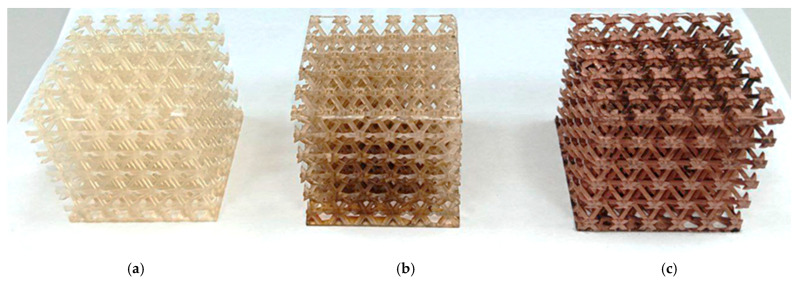
Lattice structures: (**a**) lattice structure printed from modified resin; (**b**) lattice structure immersed in an activator solution; (**c**) lattice structure with a copper coating applied.

3D printing of polymer materials was conducted using an Anycubic Photon Mono M5s photopolymer 3D printer, featuring autoleveling, fast printing speeds of up to 105 mm/h, and smart control. Samples printed from the modified resin were activated in a 0.1 M silver nitrate solution for a predetermined time. After activation, the samples were rinsed with distilled water and dried, followed by chemical copper plating using electrolytes based on Rochelle salt and trilon copper complexes. The appearance of the samples after each step is shown in Figure 1. It is recommended to pre-dry the surfaces rather than allowing them to dry freely in the air, as uneven drying can create areas where droplets accumulate, resulting in uneven coating and potential discontinuities. Additionally, insufficient drying immediately before copper plating—due to residual moisture from the introduction of the modifying additive, washing, and sensitizer treatment—leads to a loose, non-glossy copper layer with a higher tendency to peel off.

The continuity achieved during the chemical metallization of the composite material was examined using scanning electron microscopy method SEM (VEGA3 TESCAN electron microscope, Brno, Czech Republic).

The dielectric properties of the material were measured in accordance with GOST 22372-77 [52], using a Jinko JK2832 LCR universal tester (Changzhou Jinko Electronic Technology Co., Ltd., Changzhou, China) [53,54]. Measurements were performed on two sets of samples: one set comprised samples printed from pure commercial resin, and the other set consisted of resin modified with tannin.

The thickness of the material layers was measured using scanning electron microscopy. Modern, high-precision, versatile thickness gauges, such as the PCE-CT 27FN (PCE Group, Meschede, Germany) and PCE-CT 100 (PCE Group, Meschede, Germany), were also used to measure the thickness of the surface layers.

Film resistance was measured using a two-configuration four-point probe method in accordance with ASTM F1529-02 on three samples.

The measured current and voltage values were used in calculations according to the following Formulas (1)–(4) [54]:

- for values obtained using the connection diagram *A*:

(1) $$R_{a}=\frac{\left(\frac{V_{f}(23)}{I_{f}(14)}+\frac{V_{r}(23)}{I_{r}(14)}\right)}{2}$$

- for the values obtained using the connection diagram *B*:

(2) $$R_{b}=\frac{\left(\frac{V_{f}(24)}{I_{f}(13)}+\frac{V_{r}(24)}{I_{r}(13)}\right)}{2}$$

where *V_f_* and *V_r_* are the forward and reverse voltages;

*I_f_* and *I_r_* are the current values.

- the coefficient *K_a_* is calculated using the empirical formula:

(3) $$K_{a}=-14.969+25.173\left(\frac{R_{a}}{R_{b}}\right)-7.872\left(\frac{R_{a}}{R_{b}}\right)$$

- surface resistance was calculated using the formula:

(4) $$R_{s}=R_{a}K_{a}$$

To test the adhesion of copper layers to a polymer substrate, qualitative methods (quick, visual, and requiring no sophisticated equipment) are used:

1. Scotch tape method (ASTM D3359). Adhesive tape is applied to the surface of the copper film, held in place for 30 s, and then quickly removed [55].

2. Grid Cut Method (ISO 2409). Using a Konstanta KN-1 knife (Konstanta-MSK LLC, Russia), a 5 × 5 grid of cuts is made with a pitch of 1–2 mm. Tape is applied over the cuts and then removed. Adhesion is assessed on a 6-point scale, ranging from 0 (no delamination) to 5 (complete delamination) [56].

## 3. Results and Discussion

Given the tendency of tannins to coagulate in concentrated solutions and at elevated temperatures, special measures were implemented to minimize thermal exposure during the printing process. Temperature conditions were maintained at the minimum permissible level (+18 °C) during both resin emulsification and printing, in accordance with the technical requirements for the resin used. If these conditions were not met, tannin coagulated. The coagulate could appear on the sample surface, creating zones with increased reducing capacity. This led to enhanced formation of silver deposition sites from the activator solution and subsequently more active copper deposition from the electrolyte. Consequently, the copper layer exhibited areas of loose coating.

**Figure 2:**
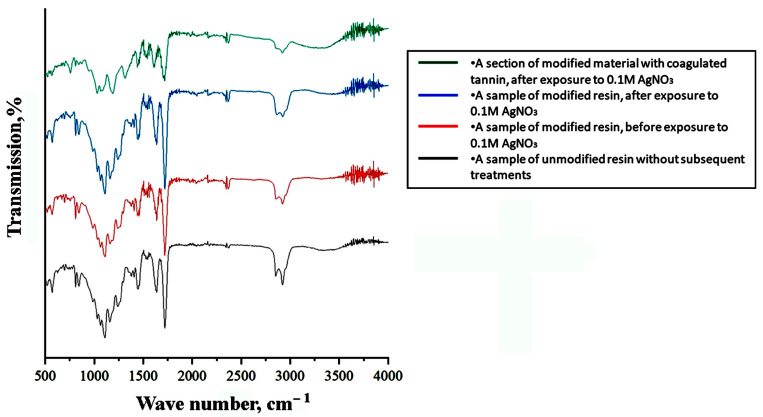
IR spectra of unmodified and modified resin samples before and after exposure to the activator, including the area of local darkening.

During the formation of the polymer composite material, IR spectroscopy was employed to monitor the chemical interactions between the resin and the modifying additive (Figure 2). To optimize the functional and mechanical properties of the composite materials, we studied polymer samples printed from unmodified resin, modified resin, modified resin followed by curing in an AgNO_3_ solution, as well as a region with tannin coagulate exposed on the surface, which resulted in a darkened area. Based on the obtained spectra (Figure 2), we concluded that there is no significant chemical interaction between the modifying additive and the base resin; their interaction is limited to the distribution of the additive within the bulk, as is typical for a composite.

Moreover, since the tannin is contained within the bulk of the product, it is not washed off the surface and does not cause damage to the AgNO_3_ solution. As it was suggested, the tannin film readily dissolves in an aqueous AgNO_3_ solution, triggering the release of atomic silver throughout the activator solution. Bulk-sensitized samples do not release tannin into the solution, and their shelf life exceeds 24 h when stored in a shaded area.

The degree of cross-linking in the polymer material can influence its ability to form deposition sites on the surface. To test this hypothesis, a series of samples were printed with varying layer exposure times of 9, 16, 23, 30, and 37 s. Shorter exposure times were not feasible due to delamination of the samples from the build platform. After printing, washing, and weighing on a VLTE-510T analytical balance (GOSMETR, Russia), the samples were soaked in 0.1 M AgNO_3_ for the same time. Following soaking, the samples were blotted to remove excess moisture and reweighed. This process is clearly reflected in the samples’ appearance, which acquires a characteristic color. The color intensity varies, as shown in Table 3.

Copper was selected as the surface layer of the composite material because its electrical conductivity is second only to gold and silver, which are significantly more expensive. Additionally, copper offers excellent adhesion and conductivity for electroplated coatings of other metals, such as nickel and silver, making it widely used in the production of various instrumentation and electronic products. A polymer base was chosen as the functional substrate due to its low cost and advanced processing capabilities—it is easier to shape using various molding technologies. However, polymers are generally inferior to other materials in both mechanical and functional properties. Applying metallic surface layers imparts conductive and shielding properties to polymer products, providing protection against photodegradation and accelerated wear. Ultimately, metallized coatings enable the rapid and cost-effective manufacturing of products that would otherwise be very expensive or even impossible to produce using metals alone.

**Figure 3:**
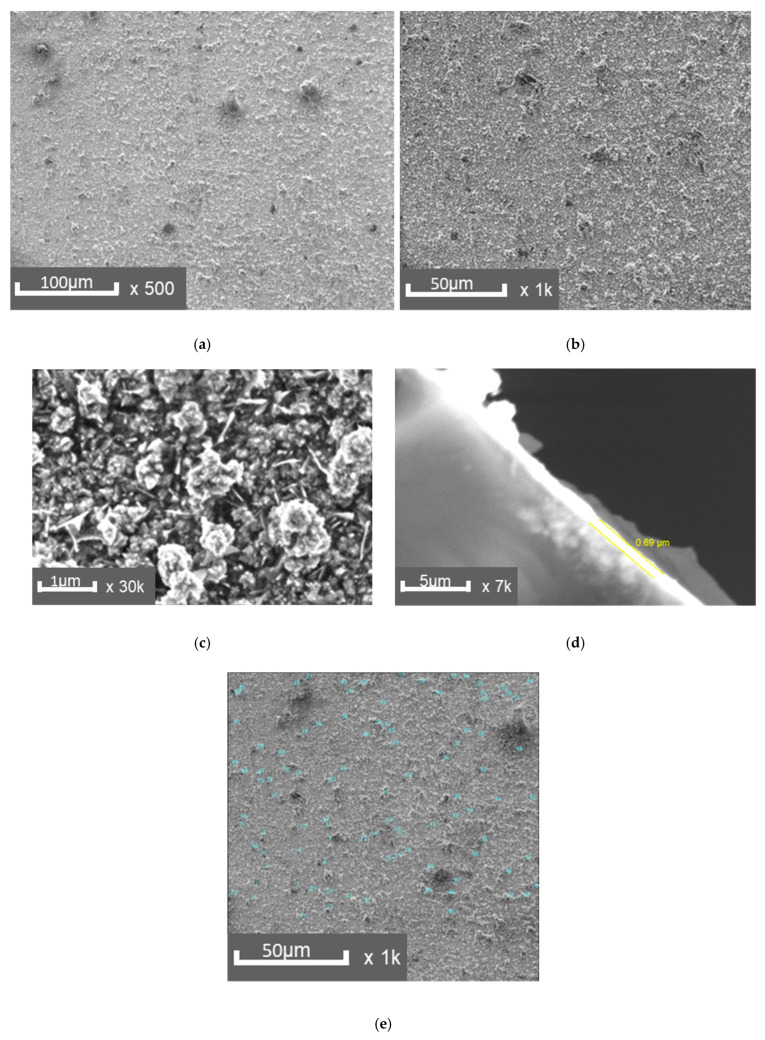
SEM images of the copper layer surface: (**a**)—×500, (**b**)—×1000, (**c**)—×30,000, (**d**)—×7000, side section of the layer, (**e**)—measurement of defects on the copper layer.

The results of a study on the structure of a polymer-copper composite material are presented in Figure 3. Macro- and microanalysis of the surface layers of the composites, produced using the established technology, revealed a sufficiently dense structure. The interface between the layers was free of visible defects (Figure 3d). The copper layer thickness ranged from 0.7 to 0.8 µm, which is optimal for the mechanical properties of the composites.

**Figure 4:**
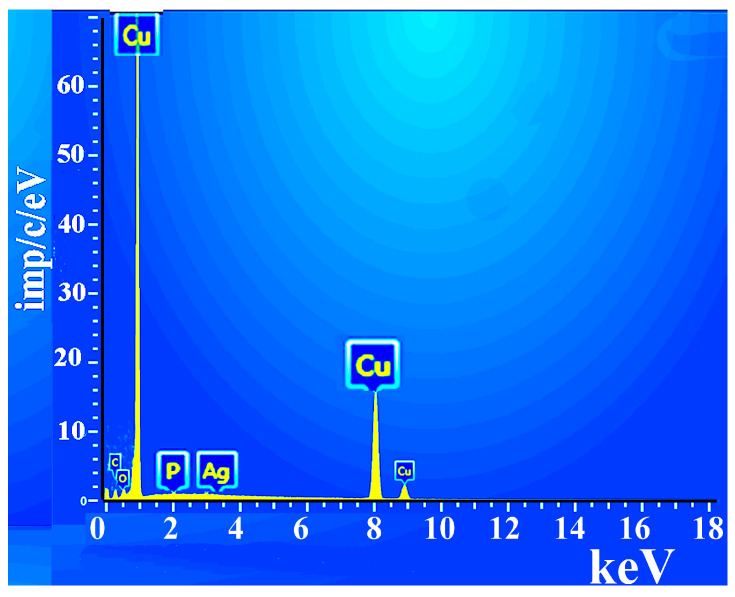
Elemental analysis of the composite surface.

A chemical analysis of the copper layer in the composite material was performed (Figure 4): Cu = 98%, C = 0.1%, O = 1.1%, P = 0.2%, Ag = 0.6%.

**Figure 5:**
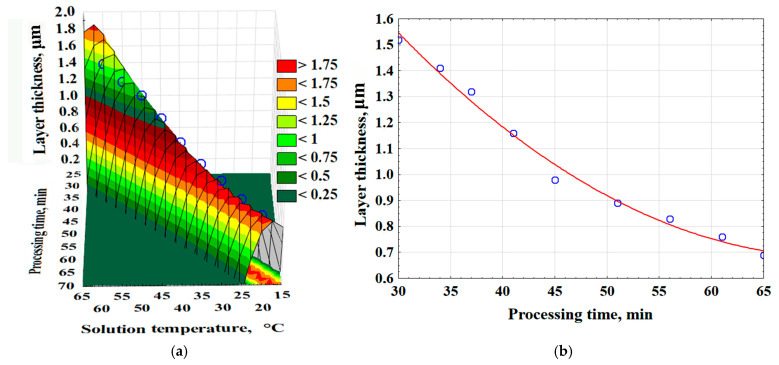
The effect of time and temperature of the solution on the thickness of the surface layer of the composite—(**a**); the effect of saturation time on the thickness of the surface layer (Red line-average values of layer thickness; Blue dots are the values obtained as a result of the experiment)—(**b**).

As a result of experimental and mathematical analysis using Statistica 10.0, a 3D model was constructed, and an empirical mathematical equation was developed to describe the effect of time and solution temperature on the surface layer thickness (δ_l_) (Equation (5)). The surface layer thickness increases more rapidly as the solution temperature rises (Figure 5).

δ_l_ = −111.8548 + 2.6313·T_s_ + 2.5637·t_p_ − 0.015·T_s_^2^ − 0.0302·T_s_·t_p_ − 0.0146 · t_p_^2^, (μm) (5)

where T_s_—temperature of the chemical metallization solution, °C; t_p_—process time, min.

### 3.1. Dielectric Properties of Materials

Dielectric parameter measurements showed that the modifying additive caused minor changes to the material’s properties. The loss tangent increased from 0.025794 (pure material) to 0.028595 (modified composition), indicating an 11.28% increase in dielectric loss. Simultaneously, the permittivity rose from 4.20371 to 4.31546 (a 2.62% increase), reflecting a change in the material’s polarization behavior. These results demonstrate that the modifying additive has a complex effect on the dielectric characteristics: it enhances permittivity while also increasing energy loss. Such changes may result from structural transformations in the material induced by the additive, which affect polarization mechanisms and relaxation processes within the dielectric. 

### 3.2. Resistance Measurement

**Figure 6:**
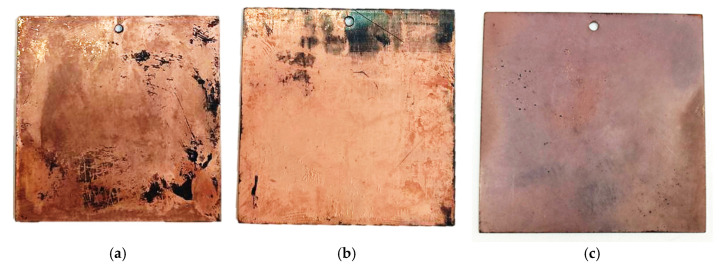
Appearance of copper-plated samples obtained in an electrolyte based on Rochelle salt (**a**,**b**) and in an electrolyte based on Trilon (**c**).

As shown in Figure 6, the layers formed in the Rochelle salt-based electrolyte exhibit a characteristic copper color. The coating is uneven, featuring shiny areas alongside redeposition zones with a looser, matte appearance. In contrast, the sample produced using the trilonate complex-based electrolyte lacks the rich red color, but has a smoother and glossier coating.

Our studies revealed significant differences in the properties of copper layers deposited from a homemade Rochelle salt-based electrolyte compared to those from a commercial Trilon electrolyte. Although the coating from our electrolyte was non-continuous, exhibiting bald spots and a reddish-copper color, the films demonstrated superior conductivity (0.056 and 0.024 Ω/□) relative to the Trilon electrolyte sample (0.091 Ω/□), which produced a continuous, glossy, but lighter-colored coating.

This paradox—the superior conductivity of a coating with less perfect morphology—can be explained by several factors. First, the composite layers obtained from the electrolyte likely have a coarser crystalline structure with fewer grain boundaries, which are the primary centers of electron scattering. Second, the commercial electrolyte likely contains nickel additives to improve adhesion, resulting in increased resistivity. Third, organic components in the commercial electrolyte (if present) could leave impurities in the film, creating additional barriers to charge movement.

Thus, the visual continuity of a composite layer does not always correlate with its electrical properties. In this case, local defects in the composite layer may be compensated for by the high purity of the copper phase and the favorable crystal orientation of the layer obtained from the electrolyte.

**Figure 7:**
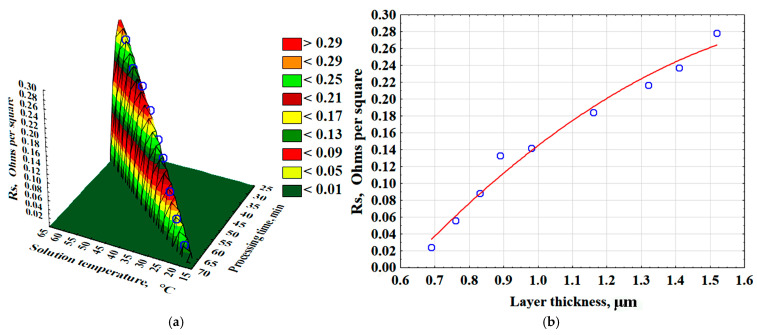
The effect of time and temperature of the solution on the resistance of the surface layer of the composite—(**a**); the effect of the thickness of the surface layer on the resistance value—(**b**).

As a result of mathematical processing of the data obtained during the experiment, a 3D model was constructed (Figure 7a). Moreover, empirical mathematical equations were developed to describe the effects of time and solution temperature on the surface layer resistance (Rs, Ω/□) (Equation (6)). The surface layer resistance increases more rapidly with rising solution temperature (Figure 7a). Additionally, as the surface layer thickness increases, the specific surface resistance also increases (Figure 7b) (Equation (7)). This parameter measures the electrical resistance of thin layers and conductors, independent of their area.

The resistance of the surface layer (Rs, Ω/□) is described by the following equations:

R_s_ = −35.1116 + 0.7575·T_s_ + 0.8595·t_p_ − 0.004·T_s_^2^ − 0.0092·T_s_ · t_p_ − 0.0053 · t_p_^2^, (Ω/□) (6)

R_s_ = −0.3226 + 0.6242 · δ_l_ − 0.1567 · δ_l_^2^, (Ω/□) (7)

where T_s_—temperature of the chemical metallization solution, °C; t_p_—process time, min; δ_l_—surface layer thickness, µm.

**Figure 8:**
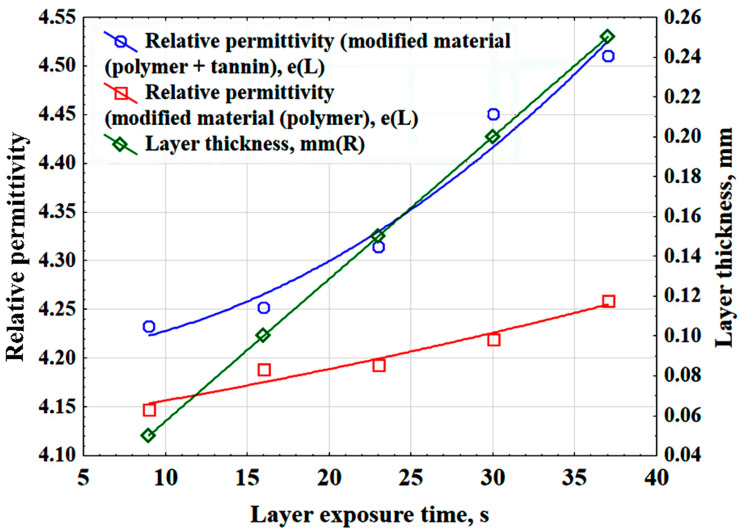
Dependences of the relative permittivity and thickness of the polymer layer on the exposure time of the layer.

By developing a 3D printing technology for polymer materials and evaluating the impact of key process parameters on electrophysical properties and layer thickness, we plotted the relationships between relative permittivity and polymer layer thickness as functions of layer exposure time (Figure 8). Layer exposure time refers to the time during which ultraviolet light illuminates the resin in the printer vat to cure and form a single layer of the printed model. The figure demonstrates that incorporating tannin into the polymer matrix increases the relative permittivity.

As a result of statistical processing of the experimental data, the relative permittivity (ε), layer exposure time (E_t_), and the thickness of the polymer layer (δ_l_^p^) are related by the following mathematical relationships:

- relative permittivity (tannin + polymer)

ε = 4.2008 + 0.0004 · E_t_ +0.0002 · E_t_^2^ (8)

- relative permittivity (polymer)

ε = 4.129 + 0.0025 · E_t_ + 2.4536 · 10^−5^ · E_t_^2^ (9)

- thickness of the polymer layer

δ_l_ ^p^ = −0.0143 + 0.0071 · E_t_ (mm) (10)

### 3.3. Evaluation of the Adhesion Strength of the Surface Layer of a Composite Material

**Figure 9:**
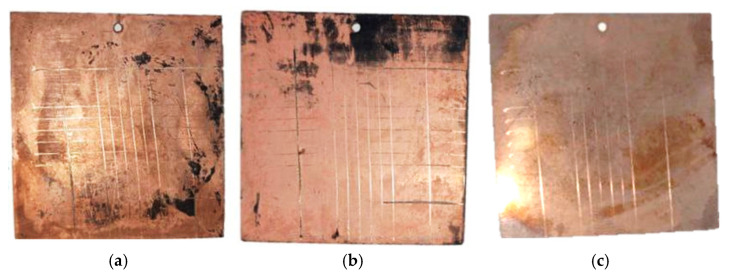
Appearance of samples after making lattice cuts with the Constant KN-1 adhesion tester knife—(**a**–**c**).

Tests of copper layers on a polymer substrate composite material demonstrated that all samples exhibited excellent surface layer durability (Figure 9). No delamination of the surface layer was observed after applying cross-hatch notches and subsequent testing, indicating a high adhesion class (0–1 according to ISO 2409 or 5B according to ASTM D3359).

Therefore, all samples meet the standard requirements, confirming the reliability of the surface layer under operating conditions.

**Figure 10:**
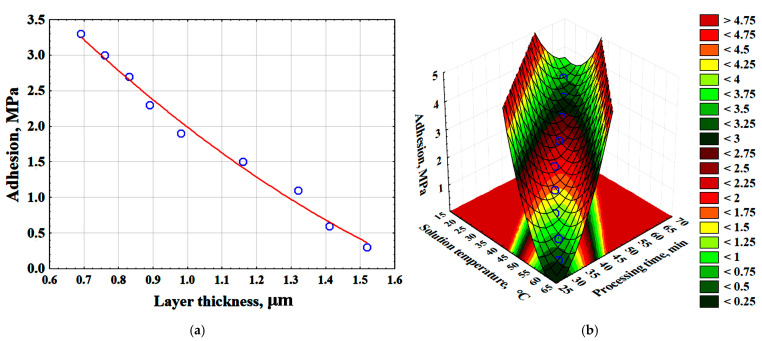
Effect of surface-modified layer thickness on adhesion (red line-average values of adhesion; blue dots are the values obtained as a result of the experiment)—(**a**); effect of time and temperature of the solution on adhesion—(**b**).

The adhesive strength of the metallic copper layer ranged from 2.1 to 2.3 MPa. Statistical analysis of the experimental data enabled the construction of graphical dependencies illustrating the effect of surface layer thickness on adhesion (Figure 10a) and the derivation of mathematical equations describing the influence of surface layer thickness (δ_l_) and key process parameters (Ts, tp) on adhesion (Equations (11) and (12)) (Figure 10b).

The adhesive strength of surface layers in functionally graded materials is described by the following equations:

S_a_ = 6.9263 − 6.1407 · δ_l_ + 1.2009 · δ_l_^2^ (MPa) (11)

S_a_ = 72.6531 − 1.6225 · T_s_ − 1.6046 · t_p_ + 0.0087 · T_s_^2^ + 0.0186 · T_s_ · t_p_ + 0.0094 · t_p_^2^ (MPa) (12)

where T_s_—temperature of the chemical metallization solution, °C; t_p_—process time, min; δ_l_—surface layer thickness, µm.

## 4. Conclusions

Technology has been developed for creating composite materials with a gradient distribution of properties, combining a polymer material (produced using SLA printing) with layers of conductive metal (applied using chemical metallization). These composites exhibit increased strength (compared to pure polymer), surface layer electrical conductivity, and increased durability, enabling the achievement of the required functional and mechanical characteristics.

As a result of technological advancements, optimal process parameters for the formation of graded materials were determined. To demonstrate the influence of key process parameters on the functional and mechanical properties of the composites, three-dimensional models were constructed and mathematical equations were developed. The influence of a modifying additive (tannin) on the dielectric characteristics of the polymer substrate was described: on one hand, the additive increases permittivity; on the other, it leads to an increase in energy loss. These changes may result from structural transformations in the material induced by the additive, which affect polarization mechanisms and relaxation processes in the dielectric. A correlation was established between the layer exposure settings and the efficiency of deposition center formation. Samples exposed for longer periods during printing exhibited lower recovery capacity, whereas samples with shorter exposure times showed more active silver deposition. This phenomenon can be exploited to selectively deposit a layer on areas printed with shorter exposure times.

SEM analysis revealed that chemical vapor deposition produces a nanocrystalline copper coating with grain sizes predominantly up to 100 nm. The morphology of the surface layer reflects controlled deposition conditions and influences its functional properties, including specific surface area and adhesion characteristics.

The electrolyte composition influenced the quality of the copper film. An electrolyte based on trilon complexes ensured better adhesion of the coating to the substrate, higher gloss, and more stable deposition compared to a Rochelle salt-based electrolyte. However, surface resistance measurements indicated that the samples prepared with the Rochelle salt-based electrolyte exhibited higher conductivity.

To determine the properties of surface conductive layers produced by the chemical metallization method, the following analyses were performed: the continuity and thickness of the copper surface layer were studied (which was found to be 0.7–0.8 μm); the dielectric properties of the polymer material were measured (relative permittivity was ε = 4.2–4.3); the resistance of the copper surface layer was measured at 0.021–0.278 Ohm/sq; and adhesion tests were conducted on the surface layer.

The obtained surface resistance values suggest that the formation of surface metal layers in functionally graded materials, using the proposed technology, can be used to create conductive structures in the electronics industry.

## Figures and Tables

**Table 1:** Main parameters of the technological process for the formation of a polymer-copper gradient material.

Layer Exposure Time (3D Printing), c	Solution Temperature (Metallization), °C	Processing Time (Metallization), min
9	25	61
16	35	51
23	45	41
30	55	34
37	60	30

**Table 2:** Composition of homemade electrolyte.

Substance	Amount in the Composition, g/L
Copper sulfate, CuSO_4_	12.5
Nickel chloride, NiCl_2_	0.01
Rochelle salt, KNaC_4_H_4_O_6_·4H_2_O	27.5
Caustic soda, NaOH	12.5
Formalin	10–15

**Table 3:** Changes in the appearance of samples and mass during the experiment.

No.	Photos of Samples		m Sample (g.) Before Aging in AgNO_3_	m Sample (g.) After Aging in AgNO_3_	Δm (%)
	Before Aging in AgNO_3_	After Aging in AgNO_3_			
TH9	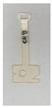	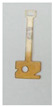	0.9596	0.9698	1.06
TH16	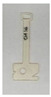	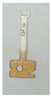	0.9452	0.9541	0.94
TH23	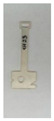	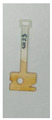	0.9507	0.9552	0.47
TH30	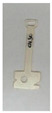	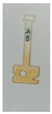	0.9688	0.9734	0.47
TH37	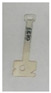	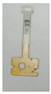	0.9953	0.9990	0.37

## Data Availability

The original contributions presented in this study are included in the article. Further inquiries can be directed to the corresponding author.
